# The influence of angiopoietin-like protein 3 on macrophages polarization and its effect on the podocyte EMT in diabetic nephropathy

**DOI:** 10.3389/fimmu.2023.1228399

**Published:** 2023-08-10

**Authors:** Yanli Ma, Yu Chen, Hong Xu, Ni Du

**Affiliations:** ^1^ Department of Pediatrics, Shanghai Fourth People’s Hospital, Tongji University School of Medicine, Shanghai, China; ^2^ Department of Nephrology, Children’s Hospital of Fudan University, Shanghai, China; ^3^ Department of Nephrology, Chongqing University Three Gorges Hospital, Chongqing, China

**Keywords:** DN, Angptl3, podocyte, macrophage polarization, NLRP3

## Abstract

**Background:**

Podocyte injury, which involves the podocyte epithelial-mesenchymal transition (EMT) process, is a crucial factor contributing to the progression of diabetic nephropathy (DN) and proteinuria. Our study aimed to examine the protective properties of Angiopoietin-like protein 3 (Angptl3) knockout on podocyte damage and macrophage polarization in DN mice and podocytes treated with HG. Furthermore, we also sought to investigate the underlying molecular mechanism responsible for these effects.

**Methods:**

DN was induced in B6;129S5 mice through intraperitoneal injection of 40 mg/kg of streptozotocin (STZ). Subsequently, the changes in renal function, podocyte apoptosis, inflammatory factors (tumor necrosis factor-α [TNF-α], interleukin-6 [IL-6], and interleukin-1β [IL-1β]), IL-10, TGF-β1, IL-1Ra, IL-10Ra, and nephrin were evaluated. Moreover, we investigated the mechanism underlying the role of Angptl3 in macrophages polarization, podocyte injury, podocyte EMT.

**Results:**

Our findings revealed that Angptl3 knockout significantly attenuated STZ or HG-induced renal dysfunction and podocyte EMT. In both *in vivo* and *in vitro* studies, Angptl3 knockout led to (1) promote the transformation of M1 type macrophages into M2 type macrophages; (2) amelioration of the reduced expression of nephrin, synaptopodin, and podocin; (3) inhibition of NLRP3 inflammasome activation and release of IL-1β; and (4) regulation of α-SMA expression via the macrophage polarization. (5) After HG treatment, there was an increase in pro-inflammatory factors and foot cell damage. These changes were reversed upon Angptle knockdown.

**Conclusion:**

Our study suggests that the knockout of Angptl3 alleviates podocyte EMT and podocyte injury by regulating macrophage polarization.

## 1 Introduction

Diabetic nephropathy (DN) is an increasingly prevalent disease worldwide, leading to end-stage renal disease and imposing a significant economic burden on society (1). Although the pathogenesis of DN is not entirely clear, recent studies suggest the involvement of immunity and inflammation in its development (2–4). While DN is not a typical immune complex-mediated renal disease, under diabetic stress, kidney intrinsic cells produce pro-inflammatory cytokines and chemokines, promoting immune response and recruitment of macrophages and mast cells (5). This leads to the activation of complement, amplification of the innate immune response, infiltration of inflammatory cells and mast cells in renal tissue and causing inflammatory injury and fibrosis of the kidney. This results in the decline of the glomerular filtration rate of renal function (6). Animal and cell studies indicate that adaptive immune cells are also involved in the process of kidney injury induced by diabetes (7). A better understanding of immune disorders and inflammatory responses is crucial in the development of new strategies for the diagnosis and treatment of DN.

Macrophages are innate immune cells that have a central role in modulating the inflammatory response against infections (8). These cells can differentiate depending on the stimuli they receive and are classified into two types: classic activated (M1) or alternatively activated (M2) macrophages (9). The classic activation pathway can be initiated by bacterial components, such as lipopolysaccharides (LPS), IFN-g, and granulocyte macrophage colony-stimulating factor (GM-CSF), which promote a pro-inflammatory phenotype, increasing the secretion of cytokines (TNF-α, IL-1β, IL-6, IL-12, and IL-18), and antimicrobial activity by producing reactive oxygen species (ROS), nitric oxide (NO), and antimicrobial peptides (10, 11). On the other hand, M2 is activated in response to IL-4 and IL-13, promoting an anti-inflammatory response by expressing high levels of IL-10 and transforming growth factor β (TGF-β), initiating tissue repair (12).

Angiopoietin-like protein 3 (Angptl3) is a secreted glycoprotein that plays a crucial role in the regulation of angiogenesis, stem cell proliferation, insulin resistance, lipid metabolism, and tumors (13–15). Its role in renal diseases, however, remains unclear. Our previous studies demonstrated that Angptl3 knockout effectively delayed the formation of glomerulosclerosis by reducing podocyte detachment and apoptosis (16–18). However, the mechanism of Angptl3 in regulating podocyte injury in STZ-induced DN mice and HG-treated podocytes is still unknown.

Based on the above studies, we hypothesize that Angptl3 knockout may alleviate podocyte EMT by promoting polarization from M1 to M2, thereby attenuating STZ-induced diabetic renal injury. This study aims to investigate the protective effects of Angptl3 knockout on renal injury and podocyte damage in DN mice and HG-treated podocytes while also identifying its molecular mechanism. Our findings support the scientific basis for Angptl3 as a new target for the prevention of DN-related renal injury and podocyte damage.

## 2 Materials and methods

### 2.1 Diabetes induction

The Angptl3-/- mice were mated with two Angptl3+/- mice and their genotypes were identified through PCR analysis, as previously described. Estrogen, a female hormone, has been reported to have a protective effect against diabetic renal injury (19), while male mice are more susceptible to STZ-induced diabetes and associated kidney damage compared to female counterparts (20). Research on regulatory effects of inflammatory response has demonstrated specific impacts of estrogen, rather than androgen (21). For these reasons, male mice were chosen for the experiment and were divided into four groups: control, DN, DN+Angptl3-/-, and Angptl3-/-, and were fed either standard (10% fat calories) or high-fat (60% fat calories) diets for 8 weeks. Mice in the DN groups (DN+Angptl3-/- and DN) were induced with daily intraperitoneal injections of STZ (40 mg/kg in 0.1 M sodium citrate buffer) for five days. Non-DN groups (control and Angptl3-/-) received equivalent amounts of citrate buffer. Mice with blood glucose levels greater than 16.7 mmol/L five days following the final STZ injection were considered successful models and were included in the experiment. Urine samples were collected at four and eight weeks following STZ injection, and blood samples and kidneys were gathered at eight weeks following injection for additional experimentation.

### 2.2 Histopathological and morphological analysis

At the end of the 8th week, the kidneys were harvested, fixed in 4% paraformaldehyde, and paraffin-embedded prior to being sectioned into 4μm slices. These slices were then stained using Masson and Periodic Acid-Schiff (PAS) staining techniques (22). Liver and pancreatic specimens also embedded in paraffin were 4 μm sectioned and stained with hematoxylin-eosin (HE) for examination. The Schmidt scoring system was used to classify and measure the histopathological changes in the pancreas, such as inflammation, edema, vacuolation, hemorrhage, and necrosis (23).

### 2.3 Bone marrow derived macrophages and cell culture

The femurs were dissected and the ends of the bone were cut. Bone marrow-derived macrophages (BMDM) were obtained by washing the bone marrow with sterile PBS and incubating with R20/30 medium for seven days. The R20/30 medium was prepared by mixing 50% RPMI-1640 medium, 20% Fetal Bovine Serum (FBS), and 30% L929 cell conditioned medium (LCCM). A fresh medium was added on the fourth day. After seven days, the macrophages were collected using ice-cold PBS, centrifuged at 1500 rpm for 5 minutes at 4°C, and then suspended in RPMI-1640 medium, LCCM, and FBS. The final composition of the suspension was 85% RPMI-1640 medium, 5% LCCM and 10% FBS. Cell counts were determined using trypan blue. The final suspension volume containing 1x106 cells per mL was distributed into four wells, 2 mL per well, for a total of 2x106 cells per well, and then incubated at 37°C and 5% CO2 for cell adhesion. After 12 hours of the incubation period, cells were primed with 100 ng/mL of LPS (Sigma-Aldrich, Saint Louis, Missouri, EUA) for 4 hours and 20 µM of Nigericin (Sigma-Aldrich, Saint Louis, Missouri, EUA) for 30 minutes. Subsequently, cells were cultured in a standard glucose concentration of 5.5 mM and a high glucose concentration of 25 mM using RPMI-1640 medium (Gibco® by Life Technologies, ThermoFisher Scientifific, Waltham, Massachusetts, EUA) and stimulated with LPS at 100 ng/mL concentration.

### 2.4 Transmission electron microscopy

Mouse kidney cortex slices were fixed first in 2.5% glutaraldehyde and 1% osmic acid for 2 hours, followed by overnight incubation at 4°C. After fixation, the samples were dehydrated, soaked in ethoxy resin overnight, and finally incubated at 60 °C for 48 hours. Ultrathin sections of 70 nm or more were obtained using an ultramicrotome and observed under an H-7500 transmission electron microscope (24).

### 2.5 Cell culture

The MPC-5 mouse podocyte cell line was obtained from Shanghai Fuheng Bio-technology Co., Ltd. (Shanghai, China). MPC-5 cells were cultured in complete RPMI1640 medium supplemented with 100 U/ml of penicillin/streptomycin, 10% FBS, and 10 U/ml of mouse interferon-γ and amplified at 33°C. To induce podocyte differentiation, cells were incubated at 37°C for 10-14 days after removal of mouse interferon-γ (25).

### 2.6 Treatment of MPC-5 cells and co-culture

Based on the given information, it seems that the experiment involves studying the effects of IL-1β and IL-10 secretion by different macrophage types (M1 and M2) on EMT (epithelial-mesenchymal transition) in podocytes. The role of Angptl3 in regulating macrophage polarization and its involvement in inducing podocyte EMT is also being investigated.

To conduct the experiment, podocytes are differentiated for 10-14 days before being exposed to different conditions for 48 hours. The different conditions include a control group with normal glucose levels, a mannitol group (as an osmotic control), a high glucose group, a high glucose group with siRNA-Angptl3 treatment, and a siRNA group with a scrambled sequence. After 48 hours of exposure to these conditions, the cells are harvested for further experiments to ascertain the effects of IL-1β secretion by M1 and IL-10 secretion by M2 on EMT in podocytes. Additionally, the role of Angptl3 in inducing podocyte EMT by regulating macrophage polarization is studied by pre-processing BMDM and dividing them into four groups. The four groups are the same as those in the previous experiment, i.e., a control group with normal glucose levels, a high glucose group, a high glucose group with siRNA-Angptl3 treatment, and a siRNA group with a scrambled sequence. Co-cultivation of MPC-5 and BDMD is done using transwells in this experiment. It is possible to further elaborate on the specific methods and techniques used in each experiment, including cell culture conditions, transfection protocols, and assays employed to measure EMT, Angptl3 expression, and macrophage polarization.

### 2.7 Immunofluorescence

Kidney tissue samples, 4 micrometers in thickness, were subject to deparaffinization and subsequent antigen retrieval processes. Renal sections were blocked with 10% bovine serum albumin for an hour at room temperature, as previously published in (26). The sections were incubated with a series of primary antibodies overnight at 4°C. These included anti-synaptopodin, anti-α-SMA, anti-NLRP3, anti-podocin, anti-TNF-α, anti-IL-1β, anti-IL-1Ra, anti-TGF-β1, anti-IL-10Ra, and anti-IL-10, each used at a dilution of 1:100. The primary antibodies were purchased from Proteintech, AiFang Biological, and Servicebio.

MPC-5 cells were first washed thrice with PBS and fixed in 4% paraformaldehyde for 30 minutes. The cells were then washed thrice with cold PBS and permeabilized for 30 minutes using 0.3% Triton X-100. Next, 10% BSA was used to block the cells for an hour. Subsequently, the cells were incubated with primary antibodies, including anti-nephrin, anti-synaptopodin, anti-α-SMA, anti-desmin, anti-NLRP3, anti-IL-1β, anti-TGF-β1, and anti-IL-10, each used at a dilution of 1:100. These primary antibodies were obtained from Servicebio, proteintech, and AiFang Biological. Following primary antibody incubation, the cells were treated with secondary antibodies. Eventually, fluorescent images were captured using a fluorescence microscope, specifically the FV3000 Olympus model.

### 2.8 Statistical analysis

For statistical analyses, Graph prism 9.0 was utilized. Normally distributed data was analyzed using the Student t-test and χ2 test, while non-normally distributed data was analyzed with the Kruskal Wallis *post-hoc* analysis. The threshold for statistical significance was set to a p value of less than 0.05.

## 3 Results

### 3.1 Angptl3 knockout attenuated M1 macrophage polarization

**Figure 1:**
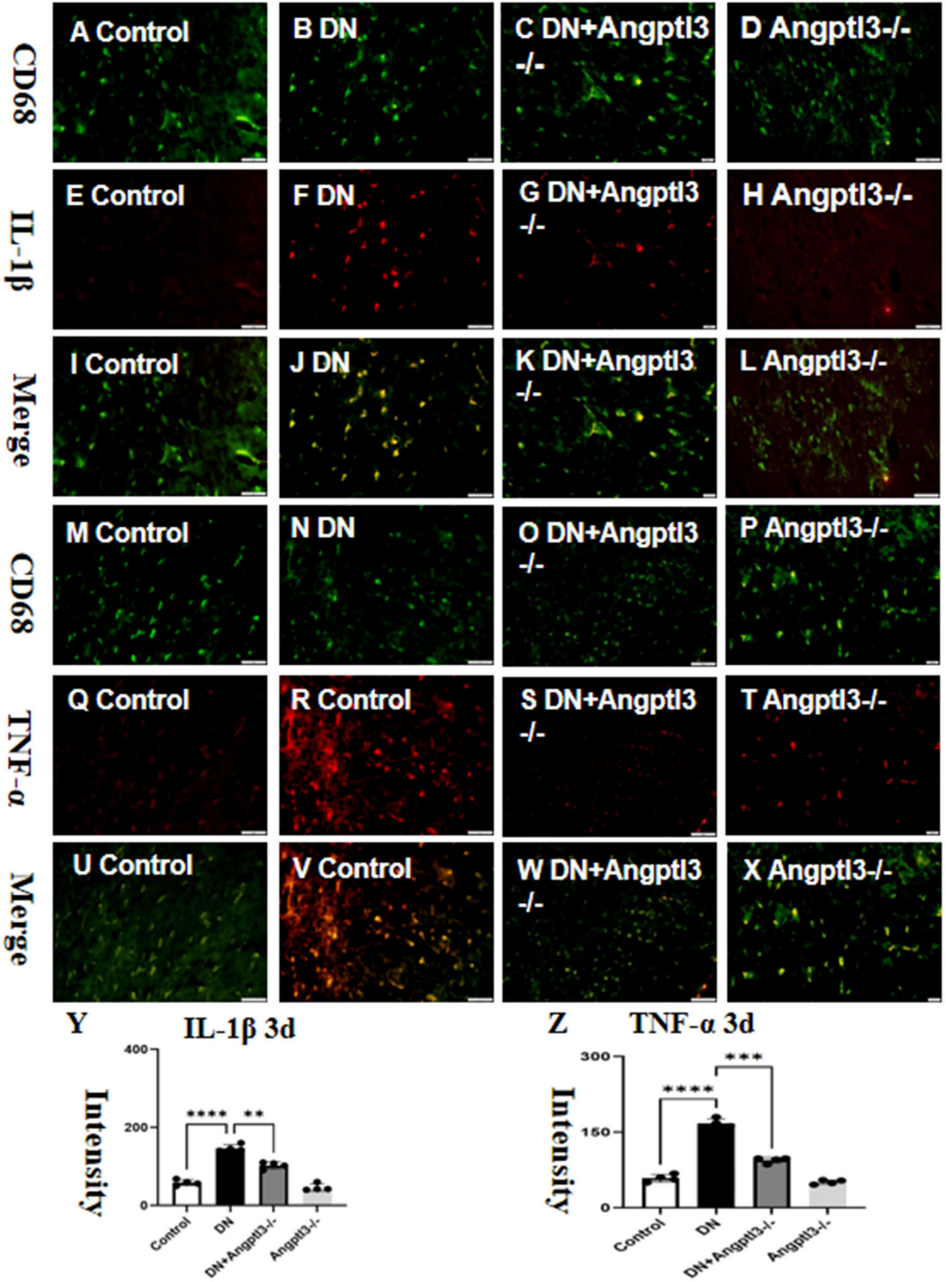
Angptl3 knockout attenuated M1 macrophage polarization. The figure illustrates the fluorescence intensity of IL-1β and TNF-α in each treatment group **(A-X)**, the expression of IL-1β was evaluated using the immunohistochemistry technique **(A-L)** and the fluorescence quantification results are shown in **(Y, Z)**. (scale bars: 50 μm for **A-X**). The sample size was four, and statistical analysis revealed significant differences (**P<0.01, ***P<0.005, ****P<0.001) between the treatment groups.

The production of Interleukin-1β (IL-1β) and Tumor Necrosis Factor-alpha (TNF-α) was significantly increased in the glomeruli of diabetic nephropathy (DN) mice. However, the production of these cytokines was reduced upon Angiopoietin-like protein 3 (Angptl3) knockout (refer to **Figures 1A–X** for more details). The Angptl3 knockout mice did not exhibit any statistically significant difference compared to the control group (refer to **Figures 1Y, Z** for more details).

### 3.2 Angptl3 knockout induced M2 macrophage polarization

**Figure 2:**
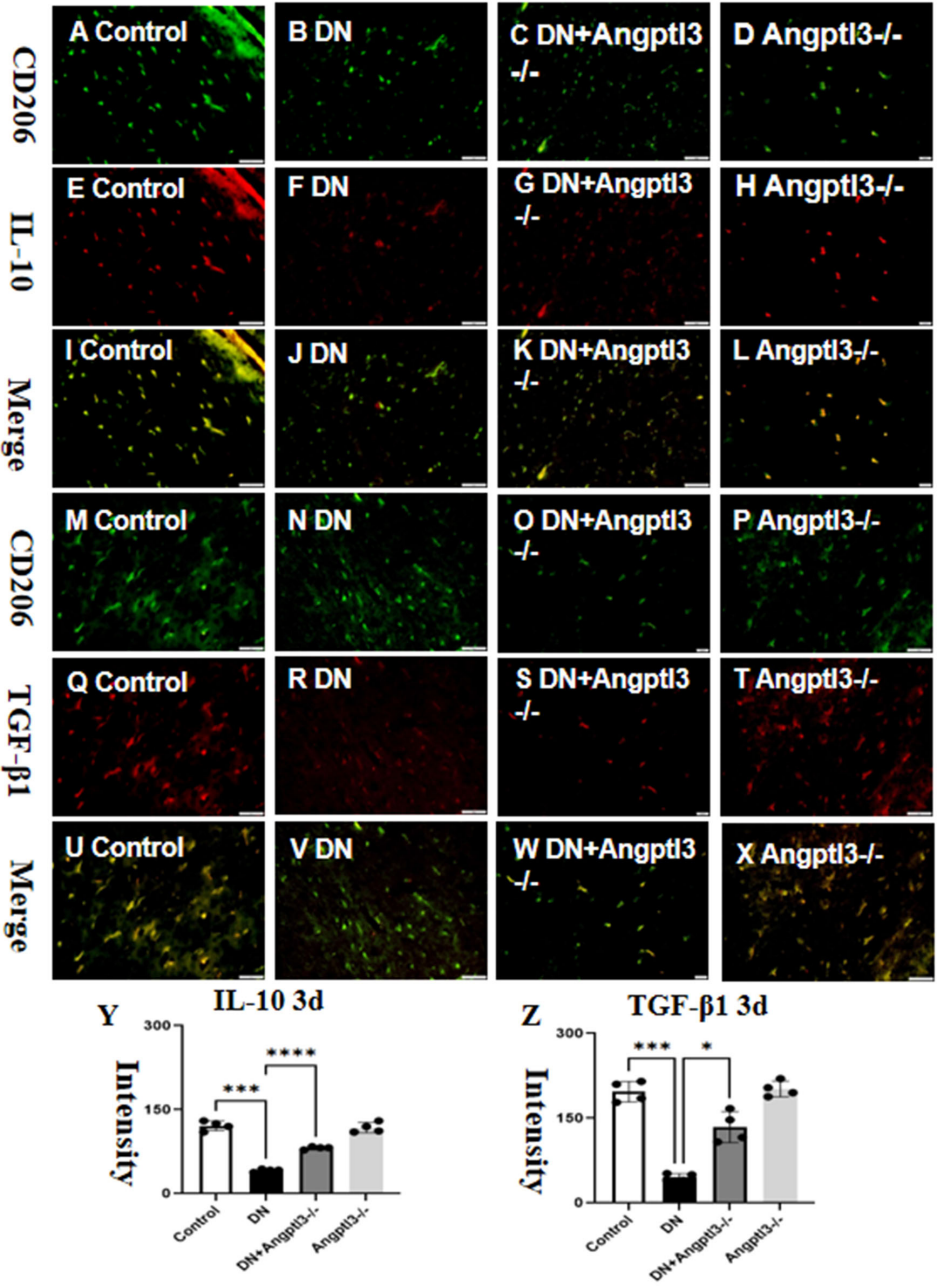
Angptl3 knockout induced M2 macrophage polarization. The distribution of IL-10 (red channel) and CD206 (green channel) in the kidney tissue was visualized using immunofluorescence (**A–H**, respectively), and the merged images of IL-10 and CD206 are presented in **I-L**. The immunoreactive figures of TGF-β1 and CD206 were presented in **(M–P)**. The bar graphs **(Y, Z)** show the quantitative analyses of IL-10 and TGF-β1 protein expression levels in each group. The sample size for the study was four, and significant differences (*P<0.05, ***P<0.005, ****P<0.001) between treatment groups were confirmed by statistical analysis. The scale bars for **(A–X)** were 50 μm.

Double-labeled immunofluorescence staining showed a significant reduction in the expression of Interleukin-10 (IL-10) and Transforming Growth Factor-beta1 (TGF-β1) in wild-type mice treated with streptozotocin (STZ) (refer to **Figures 2A–Z** for details). Protein expression levels of IL-10 and TGF-β1 were not significantly different between the control group and the Angptl3-/- group. Therefore, Angptl3 may not have a critical role in regulating the expression of these inflammatory markers.

### 3.3 There are targets for IL-1β and IL-10 on podocytes

**Figure 3:**
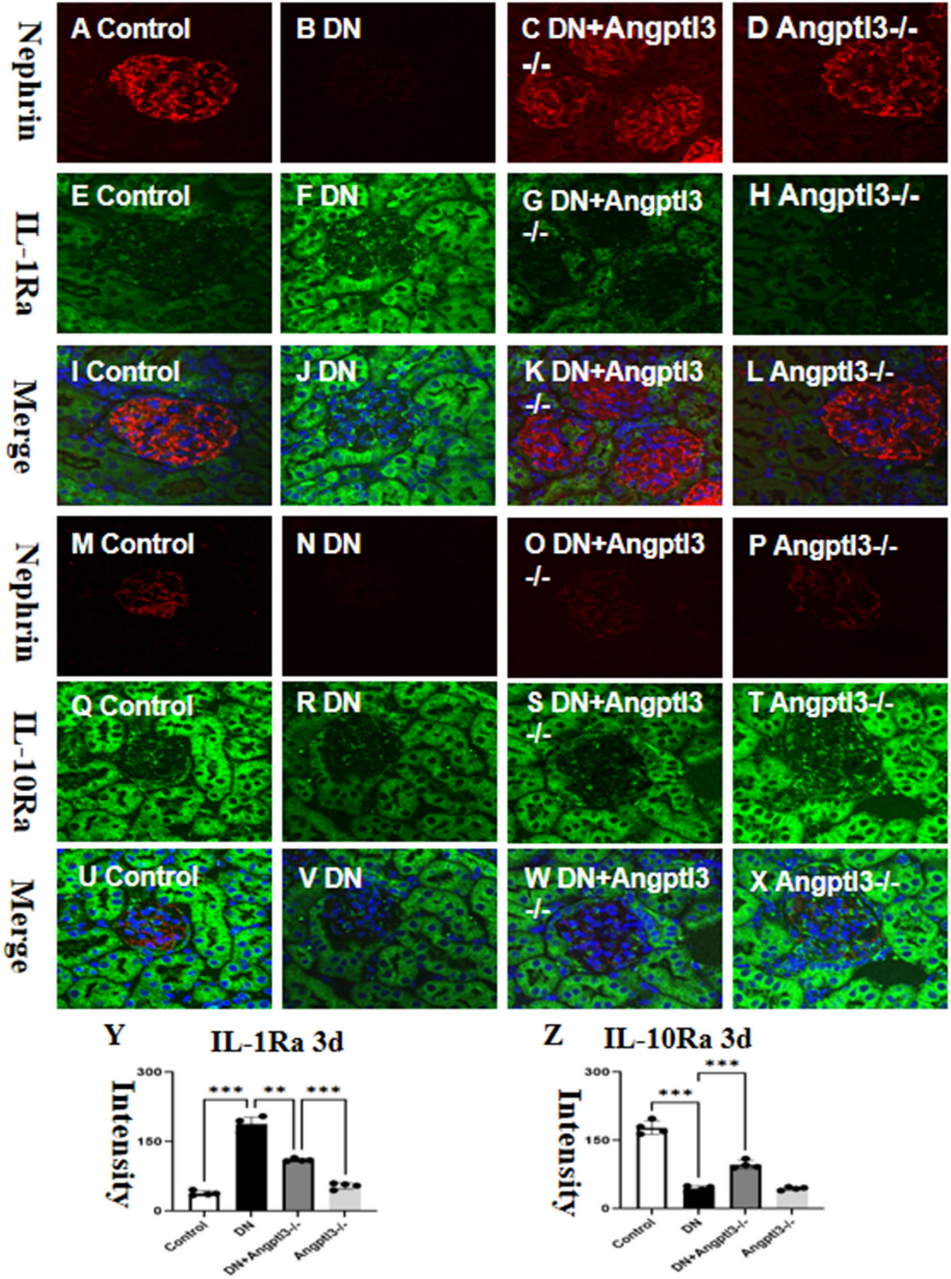
There are targets for IL-1β and IL-10 on podocytes. The distribution of nephrin (red channel) and IL-1Ra (green channel) in the kidney tissue was visualized using immunofluorescence (**A–H**, respectively), and the merged images of nephrin and IL-1Ra are presented in **I-L**. The immunoreactive figures of nephrin and IL-10Ra were presented in **(M–P)**. The bar graphs **(Y, Z)** show the quantitative analyses of IL-1Ra and IL-10Ra protein expression levels in each group. The sample size for the study was four, and significant differences (**P<0.01, ***P<0.005) between treatment groups were confirmed by statistical analysis. The scale bars for **(A–X)** were 50 μm.

Double-labeled immunofluorescence staining showed a significant upregulation in the expression of Interleukin-1 receptor antagonist (IL-1Ra) and downregulation in the expression of Interleukin-10 receptor alpha (IL-10Ra) in wild-type mice treated with STZ (refer to **Figures 3A–Z** for details). However, no statistically significant differences in the protein expression levels of IL-1Ra and IL-10Ra were observed between the Angptl3-/- group and the control group. Thus, it appears that Angptl3 does not significantly modulate the expression of these receptors.

### 3.4 The pathological changes in the glomerulus of STZ-induced DN mice were improved with Angptl3 knockout

**Figure 4:**
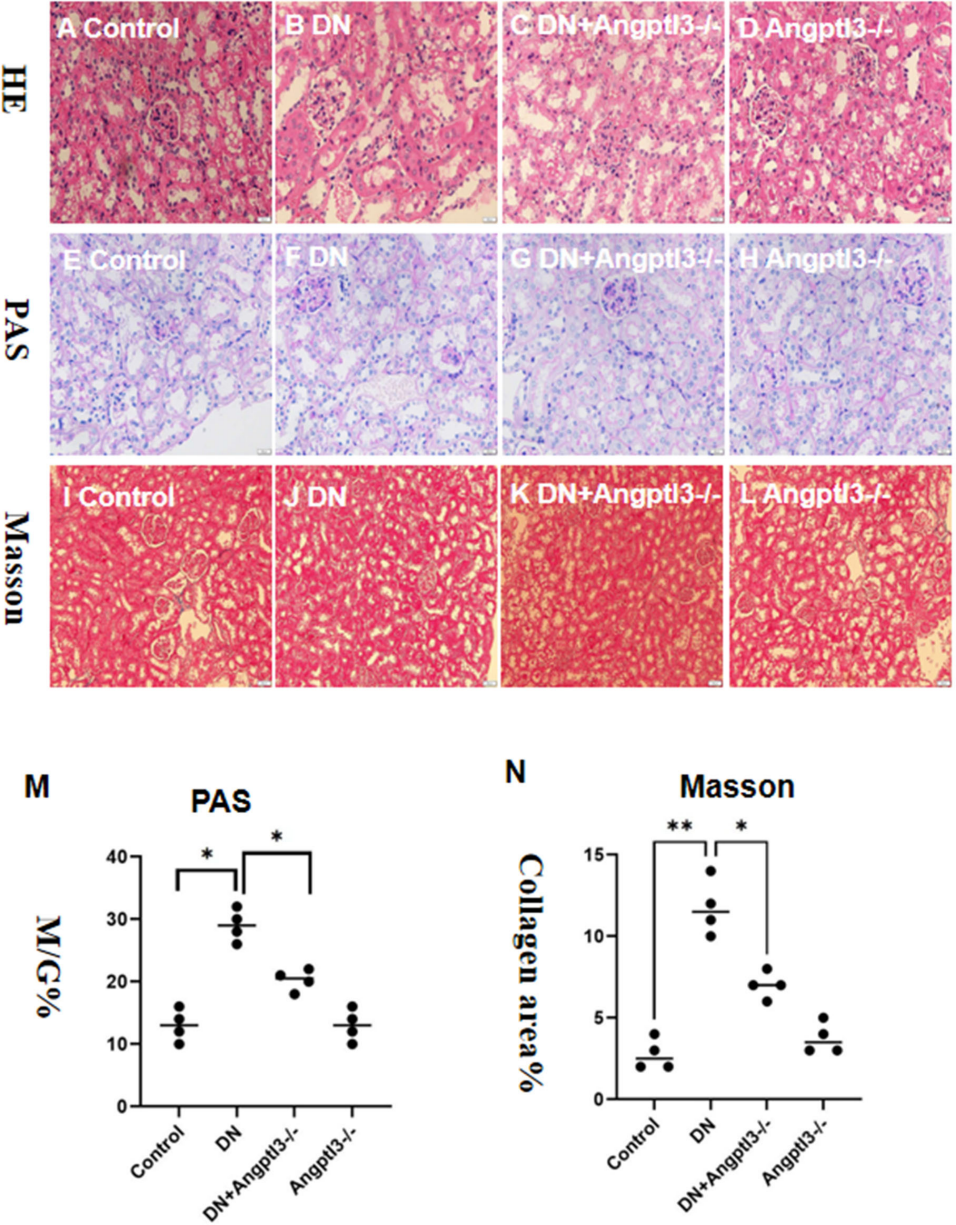
Angptl3 knockout protected the renal pathological changes of glomerulus in STZ-induced DN mice. The study includes several assessments to evaluate the effects of the treatment on glomerulus tissues. Hematoxylin and Eosin (HE) to detect steatosis, edema, ballooning degeneration **(A-D)**. PAS staining was used to examine the glomerulus under an inverted fluorescence microscope, while Masson staining was implemented to assess the degree of collagen deposition in each group **(E-H)**. The ratio of glomerular matrix area to glomerular area (M/G%) was calculated to determine the extent of matrix expansion **(M)**. Collagen area percentage was also computed to quantify the severity of fibrosis **(I-L, N)**. The sample size for this experiment was four, and the scale bars for **(A–L)** were 50 μm. *P<0.05, **P<0.01.

Glomerular capillaries of mice from the control and Angptl3-/- groups exhibited transparency, as shown in **Figures 4A, D**. Importantly, diabetic mice had more significant morphological damage in their glomeruli than the DN+Angptl3-/- group, as indicated by increased glomerular-capsule adhesions and greater capillary collapse (refer to **Figures 4B, C**). Additionally, the DN+Angptl3-/- group showed reduced collagen fiber content after undergoing Masson and PAS staining compared to the DN group (refer to **Figures 4E–N**). Consequently, these results demonstrate the protective effect of Angptl3 knockout against diabetic kidney injury (DKI).

### 3.5 Angptl3 knockout provided protection against podocyte injury in STZ-induced DN mice

**Figure 5:**
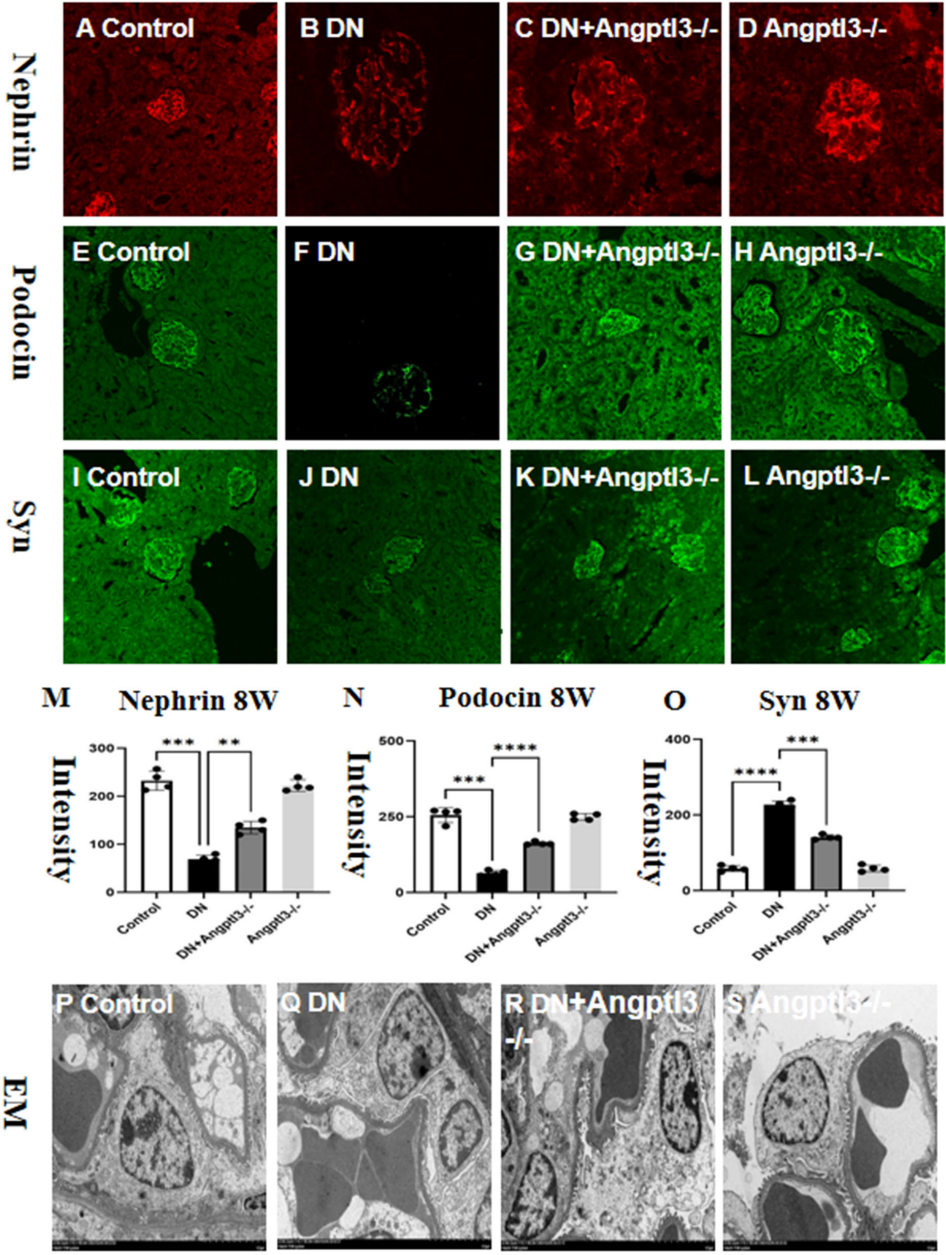
Angptl3 knockout protected podocyte injury in STZ-induced DN mice. The immunofluorescence technique was used to visualize the distribution of nephrin (red channel, **A-D**), podocin (green channel, **E-H**), and synaptopodin (green channel, **I-L**) in the kidney tissue. The fluorescence quantification results are shown in **(M-O)**. The ultrastructural changes in podocytes were assessed using electron microscopy, and the obtained images (10,000×) are presented in **(P-S)**. The sample size was four, and statistical analysis revealed significant differences (**P<0.01, ***P<0.005, ****P<0.001 between the treatment groups. The scale bars for **(A–L)** and EM were 50 μm and 1 μm, respectively.

The investigation aimed to determine the role of Angptl3 in podocyte damage in DN mice by assessing the expression levels of three podocyte markers (nephrin, synaptopodin, and podocin) in kidney tissues of each group. Immunofluorescence and Immunohistochemistry (IHC) analyses demonstrated significant decreases in nephrin, podocin and synaptopodin expression levels in glomeruli of DN mice compared to controls. Moreover, a subsequent increase in the DN+Angptl3-/- mice was observed as presented in **Figures 5A–O**. In contrast, the expression levels of nephrin, synaptopodin, and podocin did not significantly decrease in the Angptl3-/- mice as opposed to the control mice, as illustrated in **Figures 5A, E, I, D, H, L**. Control mice displayed intact and non-fused foot processes. On the other hand, DN mice showed intense fusion and effacement of foot processes, as shown in **Figures 5P–S**. Notably, Angptl3 knockout significantly improved the ultrastructure of podocytes in DN mice in terms of clear and intact foot processes with negligible fusion, as illustrated in **Figure 5Q**. Angptl3-/- mice exhibited no notable alteration in podocyte ultrastructure as depicted in **Figure 5S** when compared to the control group.

### 3.6 Angptl3 knockout mitigated podocyte EMT in STZ-induced DN mice

**Figure 6:**
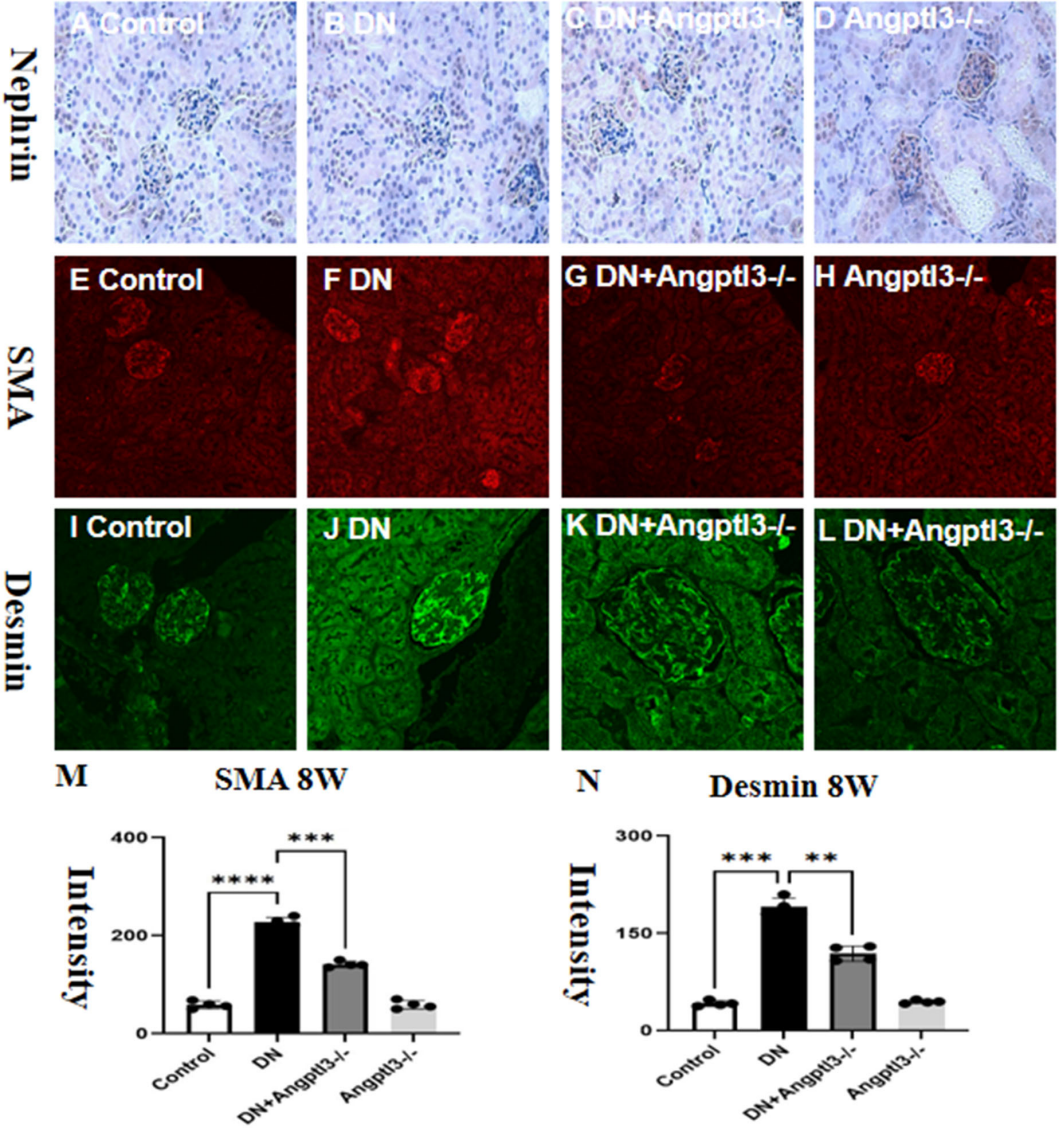
Angptl3 knockout rescued nephrin expression and inhibited α-SMA expression in renal tissue in STZ-induced DN mice. The study evaluated the protein expression levels of nephrin, desmin, and α-SMA in different groups, and the results are presented in **(M, N)**. The expression of nephrin was evaluated using the immunohistochemistry technique **(A-D)**. The distribution of SMA (red channel) and desmin (green channel) in glomerulus was visualized using the immunofluorescence technique (**E-H** and **I-L**, respectively). Bar graphs **(M, N)** show the quantitative analyses of α-SMA and desmin protein levels in each group. Statistical analyses showed significant differences (**P<0.01, ***P<0.005, ****P<0.001) between the treatment groups. The sample size was four, and the scale bars for **(A–L)** were 50 μm.

The expression of Nephrin was assessed by Immunohistochemistry. Results showed decreased Nephrin expression in the DN group, while the Angptl3 knockout group exhibited improved expression, as presented in **Figures 6A–D**. Angptl3 knockout had an inhibitory effect on podocyte epithelial-mesenchymal transition (EMT) in mice with STZ-induced DN.DN mice exhibited reduced expression levels of Nephrin protein and elevated levels of α-SMA protein compared to control mice. Conversely, Angptl3 knockout was able to counter these changes in diabetic mice, as depicted in **Figures 6E–N**. Angptl3-/- mice did not show notable Nephrin reduction nor significant elevation in α-SMA expression levels. The results imply that Angptl3 knockout can be effective in preventing podocyte EMT in diabetic mice.

### 3.7 *In vitro*, siRNA-Angptl3 promoted the activation of M2-type macrophages

**Figure 7:**
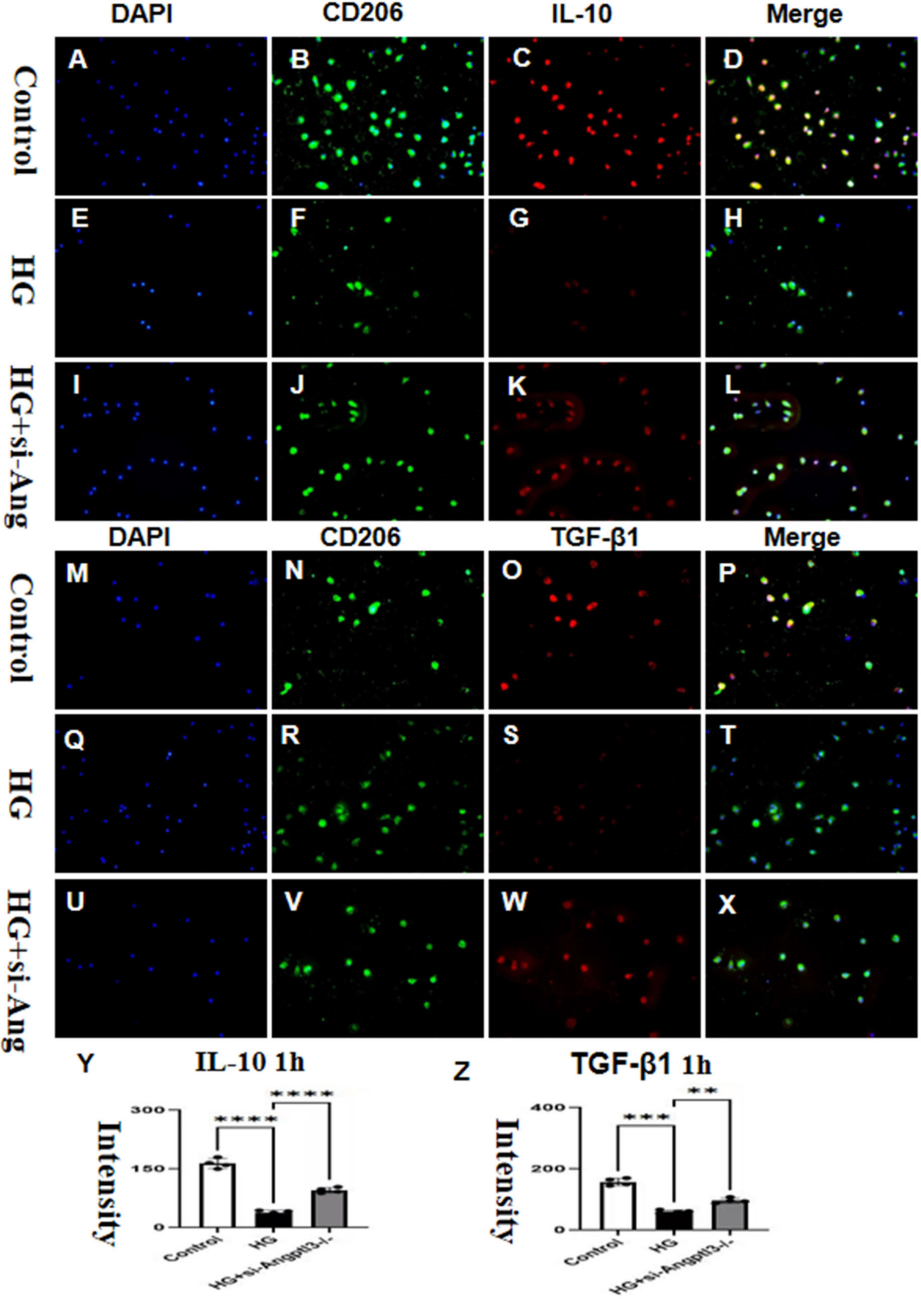
*In vitro*, Angptl3 promoted the activation of M2-type macrophages. The fluorescence intensities of IL-10 and TGF-β1 in each treatment group are presented in **(A–L, M–X)**. The quantitative analyses of IL-10, and TGF-β1 levels in each group are displayed as bar graphs **(Y, Z)**. The sample size was four, and statistical analysis revealed significant differences (**P<0.01, ***P<0.005, ****P<0.0001) between the treatment groups. The scale bars for **(A-X)** were 50 μm.

Fluorescence intensities of IL-10 and TGF-β1 in the HG group were significantly reduced when compared to the control group. The effect of HG was dampened by the knockdown of Angptl3, as revealed by **Figures 7A–X**. The expression levels of IL-10 and TGF-β1 in the siRNA group were not significantly different from those in the control group, as portrayed in **Figures 7Y, Z**.

### 3.8 siRNA-Angptl3 promotes M2 transformation and inhibits podocyte EMT by inhibiting the NLRP3 pathway

**Figure 8:**
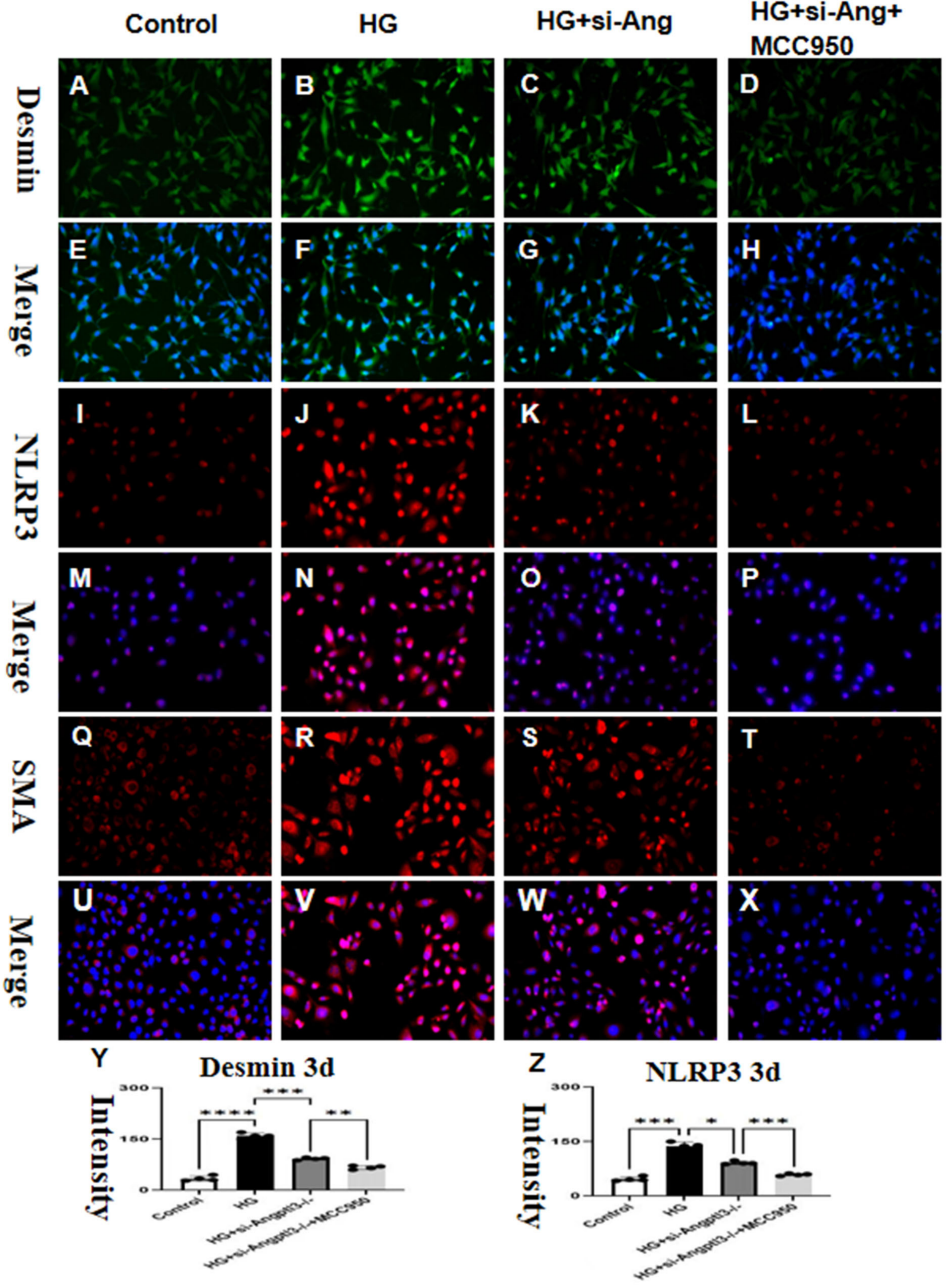
siRNA-Angptl3 promotes M2 transformation and inhibits podocyte EMT by inhibiting the NLRP3 pathway. *In vivo*, the knockout of Angptl3 yielded favorable outcomes by alleviating glomerulosclerosis and preventing podocyte EMT. This was achieved through the activation of the NLRP3 inflammasome in mice subjected to STZ-induced diabetes. In contrast, *in vitro* experiments revealed that the downexpression of Angptl3 in high glucose conditions decreased podocyte EMT which helped to curb the NLRP3 pathway **(A-Z)**. The scale bars for **(A–X)** were 50 μm. *P<0.05, **P<0.01, ***P<0.005, ****P<0.001.

The co-culture of BDMD with MPC5 diminished the impact of siRNA-Angptl3 on HG-induced podocyte injury, as depicted in **Figures 8A–Z**. HG notably upregulated the protein expression levels of desmin, α-SMA, and NLRP3, whereas their levels in HG-treated podocytes reduced significantly with siRNA-Angptl3, in comparison to the control group. We established an overexpression-Angptl3 group to elucidate the regulation of NLRP3 pathway on the expressions of α-SMA and desmin. The effectiveness of siRNA-Angptl3 was ameliorated upon the inclusion of NLRP3 inhibitor (MCC950, 10nm), whereas the converse was the case with overexpression-NLRP3, as presented in **Figures 8A–Z**. Thus, our findings imply that the NLRP3 signaling pathway mediates podocyte injury and podocyte EMT induced by siRNA-Angptl3.

## 4 Discussion

Angptl3 is a novel factor with varying expressions and functions in different diseases; however, its role in kidney diseases has been seldomly reported. Previous studies have shown that Angptl3 participates in podocyte loss induced by adriamycin, lipopolysaccharide, and puromycin aminonucleosides (PAN). Despite this, Angptl3’s role in the progression of DN has not been completely understood (27). This study found that Angpt1 genetic ablation improved podocyte injury, podocyte EMT, and M1 to M2 conversion by regulating the NLRP3 signaling pathway in DN mice. These results provide a new immune mechanism for diabetic kidney injuries and suggest that Angptl3 may be a potential preventive target for improving diabetes-related renal injuries and delaying the progression of chronic kidney diseases.

When the biological process of EMT occurs, epithelial cells transdifferentiate into mesenchymal cells, promoting pathologic fibrosis. In this process, the expressions of podocyte-specific markers, nephrin and podocin, are inhibited, whereas the expressions of mesenchymal markers, such as α-SMA and desmin, are elevated. This leads to the loss of the podocytes’ phenotype and their junction ability, resulting in hyperglycemia-mediated proteinuria. Podocyte EMT is a contributing factor to DN, renal fibrosis, and pathologic end-stage renal disease. This study found that HG-induced podocyte EMT in diabetic kidneys is characterized by increased expressions of mesenchymal α-SMA and desmin and decreased expressions of nephrin and podocin, contributing to renal fibrosis. Moreover, Angptl3 knockout reversed the pathologic damage (28, 29). Hence, Angptl3 can be proposed as a novel preventive target for attenuating podocyte EMT and pathologic renal injuries in DN.

NLRP3 inflammasome activation is responsible for HG-induced podocyte injury, according to Qiu et al. Earlier studies suggested that NLRP3 reduction or deficiency could inhibit renal inflammation and fibrosis in DN mice (30). When stimulated with HG, NLRP3 can form a multi-protein complex and interleukin-1 converting enzyme Caspase-1, which results in Caspase-1 activation. The activated Caspase-1 contributes to the secretion and maturation of IL-1β (31, 32), triggering the inflammatory cascade responses and renal injury. Therefore, the inhibition of the NLRP3 inflammasome can ameliorate DN-related podocyte injury and glomerulosclerosis. This study confirmed that NLRP3 inflammasome activation and increased expression of IL-1β participated in podocyte injury induced by HG. Angptl3 knockout inhibited the NLRP3 inflammasome and IL-1β secretion, as our previous research showed in lipopolysaccharide-induced podocyte injury. Similarly, in this study, Angptl3 knockout inhibited NLRP3 inflammasome and IL-1β secretion, thereby attenuating HG-induced podocyte injury and podocyte EMT. Hence, Angptl3 knockout’s ability to ameliorate podocyte injury and glomerulosclerosis in DN may be partially due to its anti-inflammatory action.

When cultivated in a hyperglycemic medium, BMDM secreted a significantly increased amount of TNF-α, but the expression of IL-6 was reduced, according to a study (33). Tessaro et al. reported that macrophages from various regions in diabetic animals have a dysregulated response when stimulated by LPS, impairing inflammation control (34–36). Our research found that M1 macrophages activated and released IL-1β and TNF-α under high glucose conditions. After adding siRNA-Angptl3, M2 macrophages increased and expressed IL-10 and TGF-β. We explored whether IL-10 played a mediating role in the podocytes EMT of injured podocytes, then blocked the IL-10 receptor and inhibited its expression. Inhibiting IL-10 significantly inhibited the EMT effect of HG, indicating that IL-10 was an important mediator inhibiting EMT of injured podocytes in HG. Increased IL-10 secretion from M2 cells due to siRNA-Angptl3 inhibited podocyte damage by inhibiting NLRP3 and promoting M1 to M2 transformation. Furthermore, our research showed that siRNA-Angptl3 reduces podocyte damage by inhibiting NLRP3 and promoting M1 to M2 transformation.

## 5 Conclusion

In summary, this study confirms that Angptl3 knockout is important for improving podocyte EMT in DN. It may protect against HG-induced podocyte EMT by promoting M1 to M2 polarization. These findings deepen understanding of the immunity mechanism of podocyte damage in DN and suggest that Angptl3 is a novel preventive target for HG-induced podocyte injury.

## Data availability statement

The original contributions presented in the study are included in the article/supplementary material. Further inquiries can be directed to the corresponding authors.

## Ethics statement

The institutional Animal Care and Use Committees of the Institute of Developmental Biology and Molecular Medicine of Fudan University (IDMIACUC) and Children’s Hospital of Fudan University approved the protocols for all animal experiments, under the registration number (2021) 189.

## Author contributions

ND and HX designed this study. ND and YM conducted these experiments, analyzed the data, and wrote this article. ND and YM contributed some reagents and materials. YC participated in some animal and cell experiments. All authors contributed to the article and approved the submitted version.
